# Evolution of ZW Sex Chromosomes in *Ptyas* Snakes (Reptilia, Colubridae): New Insights from a Molecular Cytogenetic Perspective

**DOI:** 10.3390/ijms26104540

**Published:** 2025-05-09

**Authors:** Príncia Grejo Setti, Tariq Ezaz, Geize Aparecida Deon, Ricardo Utsunomia, Alongklod Tanomtong, Sukhonthip Ditcharoen, Nattasuda Donbundit, Montri Sumontha, Kriengkrai Seetapan, Phichaya Buasriyot, Krit Pinthong, Weera Thongnetr, Natália dos Santos, Fábio Porto-Foresti, Thomas Liehr, Marcelo de Bello Cioffi

**Affiliations:** 1 Department of Genetics and Evolution, Federal University of São Carlos, São Carlos 13565-905, SP, Brazilgeizeadeon@gmail.com (G.A.D.); mbcioffi@ufscar.br (M.d.B.C.); 2 Institute of Applied Ecology, Faculty of Science and Technology, University of Canberra, Canberra, ACT 2617, Australia; tariq.ezaz@canberra.edu.au; 3 Faculty of Sciences, São Paulo State University, Bauru 17033-360, SP, Brazil; ricardo.utsunomia@unesp.br (R.U.); n.santos97@unesp.br (N.d.S.); fp.foresti@unesp.br (F.P.-F.); 4 Faculty of Interdisciplinary Studies, Nong Khai Campus, Khon Kaen University, Muang, Nong Khai 43000, Thailand; alotan@kku.ac.th (A.T.); sukditc@kku.ac.th (S.D.); waiinatda@gmail.com (N.D.); 5 Ranong Marine Fisheries Research and Development Center, Muang, Ranong 85000, Thailand; montri.sumontha@gmail.com; 6 School of Agriculture and Natural Resources, University of Phayao, Muang, Phayao 56000, Thailand; kriengkrai.se@up.ac.th; 7 Faculty of Science and Technology, Rajamangala University of Technology Suvarnabhumi, Nonthaburi 11000, Thailand; phichaya.b@rmutsb.ac.th; 8 Faculty of Agricultural Technology and Agro-Industry, Rajamangala University of Technology Suvarnabhumi, Phra Nakhon Si Ayutthaya 13000, Thailand; krit.p@rmutsb.ac.th; 9 Faculty of Science and Technology, Rajamangala University of Technology Krungthep, Bangkok 10120, Thailand; weera.t@mail.rmutk.ac.th; 10 Institute of Human Genetics, Jena University Hospital, Friedrich Schiller University Jena, 07747 Jena, Germany

**Keywords:** reptiles, sex chromosomes, FISH, ZW system, evolution

## Abstract

Snakes are cytogenetically dynamic, characterized by largely conserved diploid chromosome numbers although displaying varied variable evolutionary stages of their sex chromosomes. This study examined four snakes, with a special focus on the genus *Ptyas*, to provide evolutionary insights into the evolution of ZW sex chromosomes. We performed an extensive karyotype characterization using conventional and molecular cytogenetic approaches, described for the first time the karyotype of *Ptyas korros,* and revisited the karyotype descriptions of *P. mucosa*, *Chrysopelea ornata*, and *Fowlea flavipunctatus*. We found that all species except *F. flavipunctatus* have highly heterochromatic W chromosomes enriched in satDNAs or microsatellite repeats. Repetitive sequences accumulate with the heterochromatinization of the W chromosome but are not necessarily associated with this process, demonstrating the dynamic makeup of snake sex chromosomes. Autosomal locus-specific and sex chromosome probes from *Pogona vitticeps* and *Varanus acanthurus* did not show hybridization signals in *Ptyas* snakes, suggesting divergent evolutionary pathways. This finding highlighted the dynamic nature of sex chromosome evolution in snakes, which occurred independently in lizards.

## 1. Introduction

Snakes constitute one of the most remarkable classes of reptiles, with over 4000 species currently described, which are grouped into eight families, classified as caenophidians (“advanced snakes”) and non-caenophidians (“older snakes”) [1,2,3,4]. Caenophidian snakes encompass taxa such as Colubridae, whereas non-caenophidian snakes are classified under primitive clades, including the Boidae and Pythonidae families [1,5,6,7,8,9]. Snakes are known for presenting a large karyotype stability, where most species possess 34–36 chromosomes, organized into 8 pairs of macro and 9–10 pairs of microchromosomes [10,11,12,13,14,15]. Caenophidian snakes exhibit highly differentiated ZW sex chromosomes, typically the fourth or fifth largest chromosome pair, with the W chromosome frequently being heteromorphic and heterochromatic [16,17,18]. In contrast, other snake lineages often have homomorphic sex chromosomes, making them undetectable by conventional cytogenetic techniques [19]. This difficulty in identifying their sex chromosomes has led to the prevailing notion that most snakes have a ZZ/ZW system, in which females are heterogametic [6,20,21,22]. However, new evidence provides strong support for the existence of an XY system in certain lineages, suggesting that diversity in sex-determination mechanisms within the group may have been underestimated [14,21]. The co-existence of homomorphic and highly-differentiated sex chromosomes indicates a more dynamic evolutionary scenario than previously thought, reinforcing the need for more detailed molecular approaches to clarify the evolution of sex determination systems in snakes [19,21].

Colubridae represents one of the most special groups among snakes, which currently includes seven subfamilies: Natricinae, Pseudoxenodontinae, Dipsadinae, Sibynophiinae, Calamariinae, Grayiinae, and Colubrinae. Together, they make up more than half of all snake species, or about 1800 valid species [5,23]. The ecology of these snakes is notably diversified, including both venomous and non-venomous species [24,25,26,27,28,29,30]. Cytogenetic investigations of Colubridae species have revealed significant diversity in diploid numbers (2n), ranging from 2n = 24 (in *Hydrodynastes bicinctus*) to 2n = 54 (in *Clelia occipitolutea*) [17], alongside morphological changes in their sex chromosomes, including both homomorphic and a highly-differentiated ZW system [17,31,32,33]. Nonetheless, the majority of species still lack cytogenetic data, and the karyotypic diversification of numerous groups is either inadequately researched or entirely neglected [33]. The genus *Ptyas*, commonly referred to as Rat snakes, comprises 13 species primarily distributed in Southeast Asia [1], and is one of the least studied Colubridae genera. The only cytogenetic studies involving the genus *Ptyas* was carried out in the 1970s with the species *Ptyas mucosa*, which indicated 2n = 36 and homomorphic ZW sex chromosomes [31,32]. The evolutionary position of *Ptyas* within the Colubridae represents an attractive opportunity to examine patterns of repetitive sequence amplification and chromosomal evolution [34]. These analyses are contextualized through cytogenetic comparisons with other snakes from the same geographical area, albeit from different genera, or even from remote regions. This enables the analysis of the evolutionary pathways of sex chromosomes and repetitive sequences in snakes while also improving our comprehension of the processes behind chromosomal differentiation, especially the heterogeneity within the ZW sex chromosome pair.

Our study involved a thorough investigation, utilizing four snakes, with a special focus on two *Ptyas* species (the Chinese rat snake *Ptyas korros* and the Oriental rat snake *Ptyas mucosa*) as models. Our objective was to address the subsequent inquiries: (i) Are the homomorphic sex chromosomes of these snakes likewise molecularly undifferentiated? (ii) Is there a correlation between heteromorphism, heterochromatization, and the accumulation of repeated sequences? (iii) Does the W chromosome of *Ptyas* contain distinctive repetitive sequences absent on the Z chromosome? (iv) Are the same repetitions present on the Z and/or W chromosomes of *P. korros* likewise preserved on the W chromosomes of *P. mucosa* and in other closely related species? (v) Do the sex chromosomes of *Ptyas* share similar sequences with squamate lizards? To address these questions, we conducted comprehensive karyotype characterization employing both conventional and molecular cytogenetic methodologies in two *Ptyas* species, encompassing karyotype assembly, C-banding, genomic comparisons between sexes and species, Interstitial Telomeric Sequences (ITS) identification, and microsatellite sequence mapping. Subsequently, we characterized the satellitome (i.e., the whole collection of satellite DNAs) of *P. korros* and employed extensive analyses to ascertain the evolutionary trajectory of sex chromosomes in phylogenetically analogous species, encompassing other Colubridae snakes (*Ptyas mucosa*, the Yellow-spotted keelback *Fowlea flavipunctatus*, and the Golden tree snake *Chrysopelea ornata*), in addition to the Tiger snake *Notechis scutatus* (Elapidae) and the Spiny-tailed monitor *Varanus acanthurus* (Squamata, Varanidae), a member of a basal clade of lizards. Moreover, to elucidate sex sequences shared in reptiles, we also mapped sex-linked bacterial artificial chromosomes (BACs) from the Central bearded dragon *Pogona vitticeps* (Squamata, Agamidae) to examine their conservation among squamate reptiles.

## 2. Results

### 2.1. Karyotypes and C-Positive Heterochromatin

**Figure 1:**
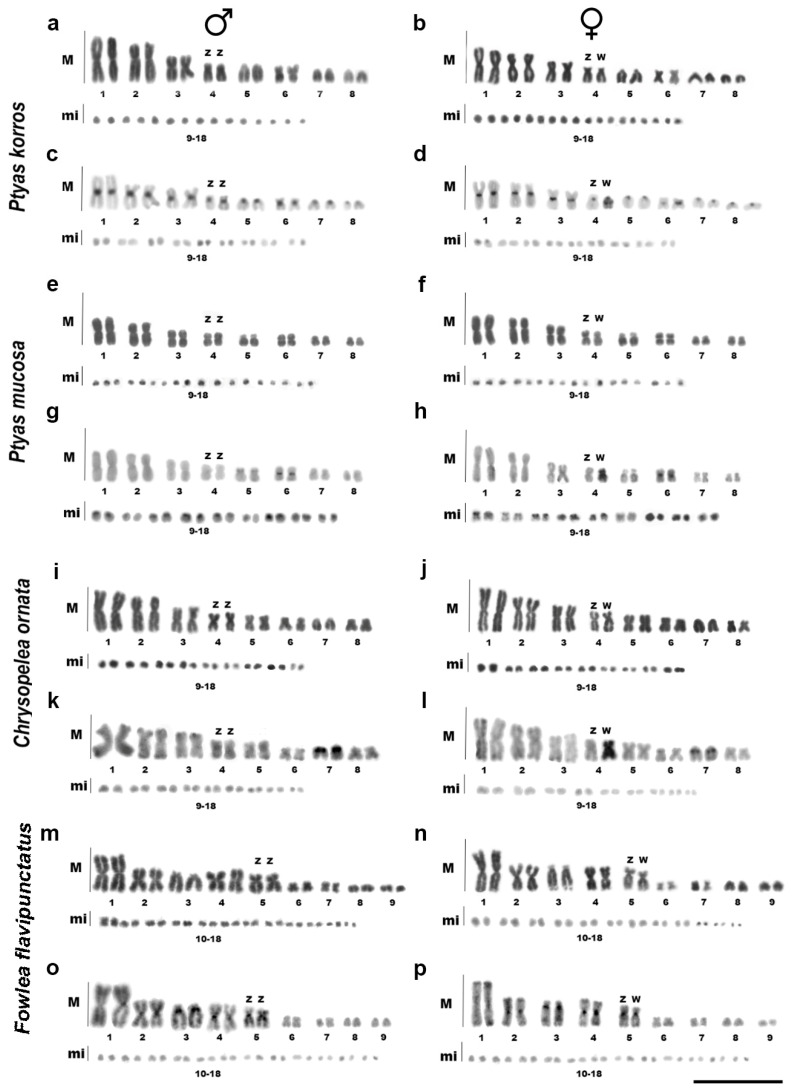
Karyotypes of *Ptyas korros* (**a**–**d**), *Ptyas mucosa* (**e**–**h**), *Chrysopelea ornata* (**i**–**l**), and *Fowlea flavipunctatus* (**m**–**p**) arranged after Giemsa staining (**a**,**b**,**e**,**f**,**i**,**j**,**m**,**n**) and C-banding (**c**,**d**,**g**,**h**,**k**,**l**,**o**,**p**). Scale bar = 10 µm.

Both *Ptyas* species (*P. korros* and *P. mucosa*) showed a diploid number of 2n = 34, with 16 macrochromosomes and 18 microchromosomes (Figure 1a,b,e,f). *C. ornata* displayed 2n = 36, with 16 macrochromosomes and 20 microchromosomes (Figure 1i,j). In contrast, *F. flavipunctatus* displayed a diploid number of 2n = 42, consisting of 18 macrochromosomes and 24 microchromosomes (Figure 1m,n).

The analysis of the general C-banding pattern revealed clear differences among the analyzed species. *P. korros* and *F. flavipunctatus* exhibited heterochromatic bands in almost all centromeres (Figure 1c,d,o,p). In contrast, *C. ornata* and *P. mucosa* showed heterochromatic marking predominantly restricted to the W chromosome, with little to no detection in other chromosomes (Figure 1g,h,k,l).

C-banding analysis further revealed a markedly heterochromatic W chromosome in both *Ptyas* species and *C. ornata*, allowing the accurate identification of the ZW sex pair as the fourth largest pair in the karyotype of these species (Figure 1d,h,l). Despite minor variations in the centromeric location of the W chromosome, the ZW sex chromosomes were indistinguishable after Giemsa staining. Only *F. flavipunctatus* exhibited no accumulation of heterochromatin in the W; nonetheless, the significant heteromorphism between the Z and the W facilitated the accurate identification of the sexual pair as the fifth largest pair in the karyotype in this species (Figure 1n,p). In addition to the analyses, microsatellites were also used to identify the sex chromosomes (to be described below).

### 2.2. Satellite DNA Content of Ptyas korros and Their Chromosomal Location

We uncovered six satellite DNA families in the *P. korros*’ genome, hereafter referred to as PkoSatDNAs (see Table S1). The RUL had a median of 167 base pairs and ranged from 149 to 324 base pairs. The percentage of adenine and thymine was 63.09%, reflecting a predominance of these base pairs. Figure S1 shows the repeat landscapes, which illustrate the distribution and variation between all the PkoSatDNA families. The complete results for each satellitome are described in Table S1. Sequences are available on the NCBI-Genbank, under the accession numbers PV358982–PV358987

**Figure 2:**
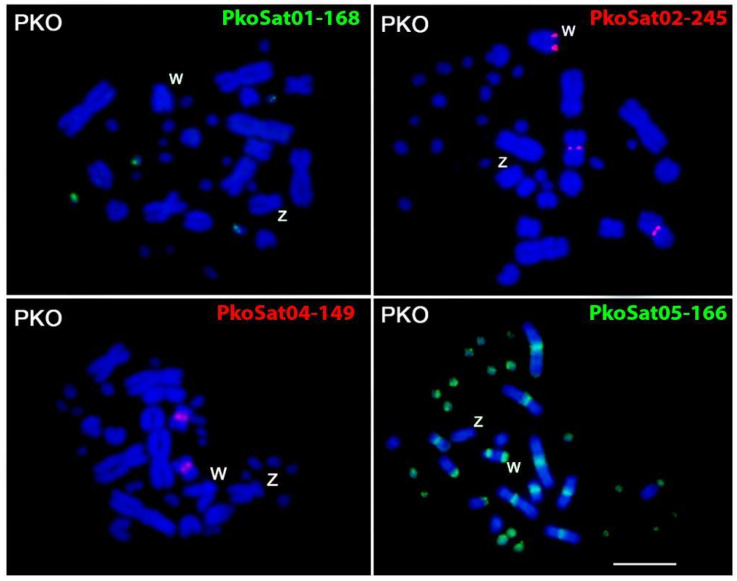
Female metaphase plates of *Ptyas korros* (PKO) hybridized with different PkoSatDNAs. Scale bar = 10 µm.

Although all six PkoSatDNAs were successfully amplified by PCR (Figure S2), only four of them (PkoSat01-168, PkoSat02-245, PkoSat04-149, and PkoSat05-166) displayed positive hybridization signals on the chromosomes of *P. korros* (Figure 2). PkoSat01-168 and PkoSat04-149 were found on autosomes, with PkoSat01-168 hybridizing on two pairs of microchromosomes and PkoSat04-149 on one pair of macrochromosomes (Figure 2). PkoSat02-245 was present in the subtelomeric region of the W chromosome and the centromere of the third longest pair of autosomes (Figure 2). PkoSat05-166 was mapped in the centromeric regions of almost all chromosomes, except for the W, which also showed signals mainly in the telomeric region of the long arms (Figure 2). No signal was detected on chromosome Z.

### 2.3. Amplification of PkoSatDNAs in Other Species

Within the PkoSatDNAs, only PkoSat01-168, PkoSat02-245, PkoSat03-324, and PkoSat05-166 were detected in the *P. mucosa* genome. Furthermore, only PkoSat02-245 and PkoSat04-149 were identified in the *V. acanthurus* genome (see Figure S2). No indication of PCR amplification of any PkoSatDNA was seen in the other examined species. Nonetheless, additional FISH analyses revealed no evidence of hybridization on the chromosomes of any species, except *P. korros* (Figure 2).

### 2.4. Chromosomal Mapping of (TTAGGG)n and Microsatellite Motifs

To investigate patterns of chromosomal differentiation, particularly in the sex chromosomes, we performed fluorescence in situ hybridization (FISH) using both microsatellite and telomeric sequences as probes. Previous studies have shown that certain microsatellite motifs—such as (AG)n, (AAGG)n, (AAT)n, (AGAT)n, and (ATCC)n—often accumulate on degenerated sex chromosomes, especially the W chromosome, in various vertebrate groups [35,36]. These motifs can serve as indicators of structural differentiation and may help reveal evolutionary processes acting on sex chromosomes. Additionally, the telomeric repeat probe (TTAGGG)n was used to assess the integrity of chromosome ends and to identify possible interstitial telomeric sites (ITSs), which can indicate past chromosomal fusion or intrachromosomal rearrangement events.

**Figure 3:**
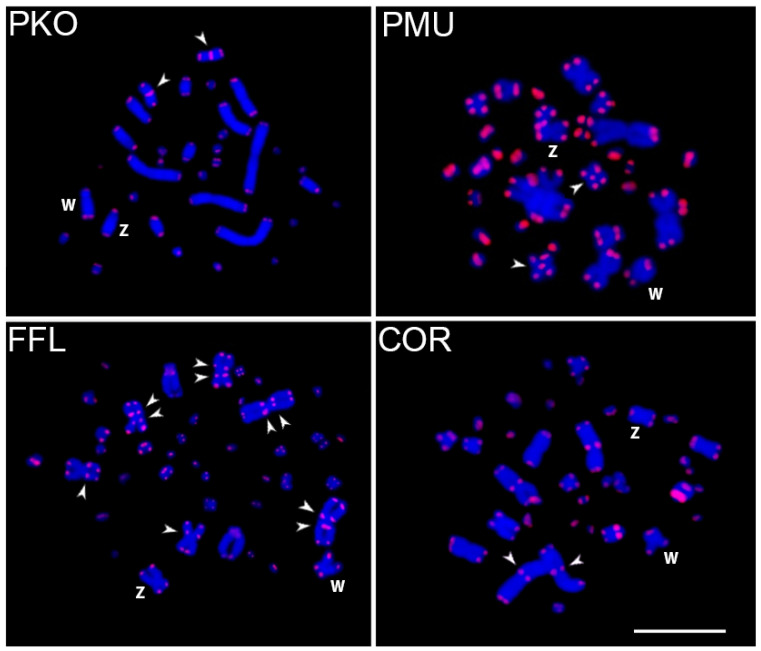
Female metaphase plates of *Ptyas korros* (PKO), *Ptyas mucosa* (PMU), *Fowlea flavipunctatus* (FFL), and *Chrysopelea ornata* (COR) hybridized with a telomeric (TTAGGG)n probe. The arrowheads indicate the interstitial telomeric sequences (ITSs). Scale bar = 10 µm.

Telomeric (TTAGGG)n repeats exhibited ITS in all examined snake species: (i) on the sixth pair of chromosomes in both *Ptyas* species, (ii) on the first pair in *C. ornata*, and (iii) on the first three pairs in *F. flavipunctatus* (Figure 3). However, no ITS was detected in the sex chromosomes.

**Figure 4:**
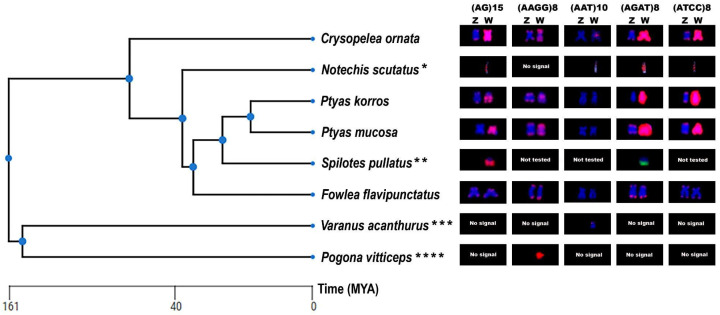
Truncated phylogenetic tree highlighting hybridization patterns of microsatellite repeats. The images referring to the W chromosome of *Notechis scutattus* and (*) *Pogona vitticeps* (****) were taken from the study published by [36] while the W chromosomes of *Spilotes pullatus* (**) and *Varanus acanthurus* (***) were taken from the studies of [33] and [35], respectively.

The chromosomal distribution of microsatellite repeats revealed varied hybridization patterns among the ZW sex chromosomes of the species analyzed. Three sequences, (AG)n, (ATCC)n, and (AGAT)n, showed massive accumulation on the W chromosome in both *Ptyas* and *C. ornata*, displaying an intense signal along its entire length (Figure 4). The (AAGG)n motif showed a dispersed distribution along the W chromosome of *C. ornata*, while in *P. korros* it was concentrated in the centromeric region, and in *P. mucosa* it showed a bitelomeric pattern. The motif (AAT)n, on the other hand, accumulated exclusively in the centromeric region of the W chromosome of *C. ornata*. In general, the autosomal chromosomes showed predominantly telomeric or centromeric marking patterns. The Z chromosomes showed similar marking patterns to the autosomes, except the (AGAT)n motif in *P. korros*, which showed no signs (Figure S3). In *F. flavipunctatus*, the microsatellite motifs analyzed were restricted to the telomeric regions, with no distinction between sex and autosomal chromosomes (Figure S4). In addition, the (AT)n sequence was absent, as observed in species of the genus *Ptyas*.

The W chromosomes were identifiable due to their significant heterochromatic accumulation (see Figure 1) and the differential accumulation of microsatellites. In both *Ptyas* and *C. ornata*, the W exhibits a significant signal for the (AGAT)n, (AG)n, and (ATCC)n sequences (Figure S3).

To complement our analyses, we constructed a phylogenetic tree using the TimeTree platform, incorporating our own data along with previously published cytogenetic data for *S. pullatus* [33], *N. scutatus*, *P. vitticeps* [36], and *V. acanthurus* [35]. These species were included to provide comparative context for our findings (Figure 4).

### 2.5. Comparative Genomic Hybridizations

**Figure 5:**
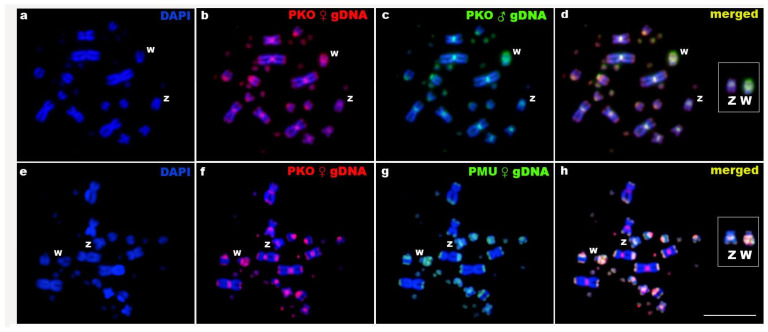
Female mitotic metaphase plates of *Ptyas korros* (PKO) after intra (**a**–**d**) and inter (**e**–**h**) comparative genomic hybridization analyses. (**a**,**e**) DAPI images; (**b**,**f**) hybridization patterns of PKO female genomic probes; (**c**) hybridization pattern of PKO male genomic probe; (**g**) hybridization patterns of *Ptyas mucosa* (PMU) female genomic probes; (**d**,**h**) merged images of both genomic probes and DAPI counterstaining. Regions stained equally by both compared genomic probes are depicted in yellow (i.e., a combination of green and red). The ZW sex chromosomes are boxed. Scale bar = 10 µm.

We first performed intraspecific comparisons primarily to examine the molecular makeup of the sex chromosomes of *P. korros*. Through the hybridization of male and female genomic DNA in metaphase plates of females from *P. korros*, all chromosomes (except the W) stained equally with these probes, mostly in the centromeric regions (which are highly heterochromatic, as shown by C-banding) (Figure 1c,d). The female whole genomic DNA probe painted the whole W chromosome, with a very bright signal on most of the Wq (Figure 5a–d). Both female and male gDNA probes showed strong hybridization to the W chromosome, as illustrated in Figure 5c. This pattern suggests the presence of repetitive sequences shared between the centromeric region of the Z and most of the W chromosome, with a possible amplification of these elements on the latter. Although CGH can detect broadly distributed repetitive elements, this hybridization pattern was not observed with any of the other probes tested in this study. Thus, this result highlights a potentially unique and previously unrecognized feature in the differentiation of the W chromosome in this species.

In the interspecific investigation, no substantial variations in the genomic composition of the W chromosomes of the two *Ptyas* species were observed. However, we observed the accumulation of *P. korros*-specific sequences in the centromeres of several autosomes (Figure 5e–h).

### 2.6. Sex Chromosome Homology Between Snakes and Lizards

In bearded dragon *P. vitticeps* (PVI), the BAC clone-designated pv3L7 hybridizes particularly to the telomere of the long arms of chromosome 2 and with one telomere of each of the Z and W microchromosomes, as previously reported by [37] (Figure S5). Conversely, the BAC clone pv116G15, which encompasses the NRSA1 gene—crucial for sex differentiation—also hybridizes with the Z and W sex chromosomes, but at the telomere opposite to the binding location of the pv3L7 sequence on each chromosome (Figure S5). No hybridization signals of the examined sequences were observed in any snake species.

To complement our investigation of the sex-linked sequences in lizards and snakes, we employed probes previously developed by [38] from the W and Z chromosomes of *V. acanthurus*.

In *V. Acanthurus*, the W chromosomal probe displayed signals on both the W chromosome and the telomere of the long arms of a macrochromosome pair (Figure S6). Conversely, the Z chromosome probe displayed a strong signal on the Z in addition to faint signals in the W and in three pairs of microchromosomes(Figure S6). Both results confirm previous findings [38]. However, when hybridized in the two *Ptyas* species and *P. vitticeps* they did not exhibit any positive hybridization signals.

## 3. Discussion

This work answers numerous critical issues about snake sex chromosomal differentiation, which we presented at the end of the introduction section. We demonstrated that the sex chromosomes of these four snake species are homomorphic yet show some degree of molecular differentiation. We also identified a high correlation between heterochromatization and repetitive repeat accumulation, notably on the W chromosome, suggesting these sequences are critical for its differentiation. We found that the W chromosome of *P. mucosa* has distinct repetitive sequences from the Z and confirmed that several of the repeated sequences found in *P. korros*’ W chromosomes are also found in *P. mucosa*, showing that closely related species share similar sequences but with some variation in abundance and distribution. These findings allow us to shed light on how snake sex chromosomes evolve and differentiate.

### 3.1. General Karyotypic Organization of Colubridae

In this study, we focused on analyzing the satDNA content of the Chinese rat snake *P. korros* (which exhibits a homomorphic ZW sex chromosome system) and exploring the molecular composition of the W chromosome concerning its evolutionary trajectory in phylogenetically analogous species, encompassing other Colubridae and Elapidae snakes and a basal lizard (*V. acanthurus*). Besides elucidating the molecular composition of the W chromosome in two underexplored *Ptyas* species, the results indicated that the accumulation of repetitive sequences is directly linked to the heterochromatization of the W chromosome, although it is not consistently associated with its heteromorphism. This finding highlighted the dynamic nature of sex chromosome evolution in snakes, which occurred independently in lizards.

Among snakes, 2n = 34 and 2n = 36 chromosomes represent the two most common diploid numbers, with variants ranging from 2n = 24 to 2n = 52 observed in various families [10,17,39]. In this work, we described for the first time the karyotype of the colubrid snake endemic to southeast Asia, *P. korros* (2n = 34, composed of 16 macro and 18 microchromosomes), and revised the karyotypes of the other three species, all cooroboratinging their previous descriptions: *Ptyas mucosa* (2n = 34) [31,32,40], *Chrysopelea ornata* (2n = 36) [40,41], and *Fowlea flavipunctatus* (2n = 42) [42]. The diploid number variation found here agrees with the range of 2n most generally reported in snakes, except for *F. flavipunctatus*, which exhibits a higher 2n than typically seen in other Natricinae species [10,17].

In our study, interstitial telomeric sites (ITSs) were detected in all analyzed species, a pattern consistent with findings in other snake lineages such as Boidae, as reported by [6,14]. However, some species within the Colubridae family have been reported to lack ITSs, indicating that the presence of these elements can vary significantly among groups [33]. This variability suggests that, although squamate reptiles generally exhibit conserved karyotypes, they may experience a relatively high rate of intrachromosomal rearrangements and a low rate of interchromosomal rearrangements [6].

### 3.2. Genomic Composition and Organization of Satellite DNA in Ptyas korros and Its Shared Characteristics with Snakes and Lizards

Snakes represent an intriguing group for genomic and cytogenetic investigation, particularly concerning the variety of repetitive sequences in their diversification and evolution [43]. Significant progress has been made concerning satellite DNA investigations within vertebrate genomes, particularly in birds [44,45,46,47,48,49,50,51], mammals [52,53,54,55,56,57], reptiles [58,59,60], and fishes [61,62,63,64,65]; however, knowledge regarding these sequences in snakes remains limited to a few species, the majority using only classical methods of satellite isolation [32,66,67,68,69,70].

**Figure 6:**
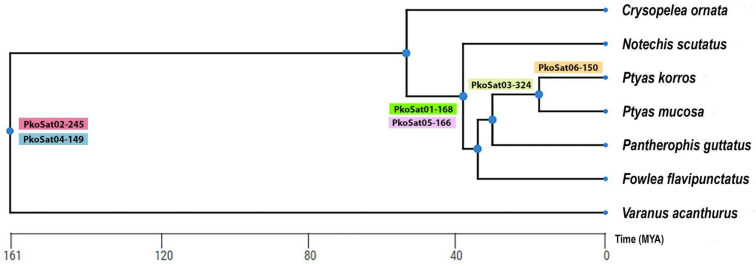
Phylogenetic tree of analyzed species obtained using TimeTree (http://www.timetree.org, accessed on 10 February 2025) highlighting the putative origin of PkoSatDNAs.

In this context, snake genomes challenge the widely accepted paradigm of a linear correlation between genome size and repetitive sequence content, as their genome sizes range from 1.3 to 3.8 Gbp, and may exhibit variable levels of repetitive elements [43,71,72,73]. We uncovered six satDNA families in *P. korros* and our *in silico* analysis demonstrated significant similarities between two PkoSatDNAs and two other satDNAs derived from *Pantherophis guttatus* (Colubridae), (hereafter designated as PguSatDNAs), previously documented by [74]. PkoSat01-168 and PguSat03-169 show an identity of 93.49%, whereas PkoSat05-166 and PguSat01-167 revealed a similarity of 81.44%.Both variants had similar chromosomal distribution across their respective species, including the pattern observed on the W chromosome, suggesting a common ancestral origin and subsequent differential amplification of these sequences throughout Colubridae. By integrating in silico, in situ, and PCR analyses, we inferred a possible evolutionary pathway for the origin of the PkoSatDNAs across selected snake and lizard species (Figure 6).

Our study suggests that PkoSat02-245 and PkoSat04-149 appear to have evolved from a common ancestor of snakes shared by *V. acanthurus* used in this study. Although these sequences remained minimally amplified in *P. mucosa* and *V. acanthurus*, they experienced a significant evolutionary expansion in *P. korros*, a scenario that suggests a divergence in the amplification mechanism among species. In contrast, these same sequences seem to have been eliminated in other snakes of the same group. Moreover, the PkoSat01-168 and PkoSat05-166 sequences originated from a common ancestor of the *Ptyas* species, *N. scutatus, P. guttatus,* and *F. flavipunctatus*. We noted considerable amplification of these sequences in *P. korros* and *P. guttatus*; however, in *P. mucosa*, the amplification was less pronounced, and in *F. flavipunctatus* and *N. scutatus*, these sequences seem to be lost. This pattern suggests that the mechanisms governing the evolution of certain satDNA sequences differ among lineages. PkoSat03-324 seems to be widespread across other *Ptyas* species, but PkoSat06-150 is exclusive to *P. korros*. The sharing of ancestral sequences, displaying differing degrees of amplification among related species, is a phenomenon well-documented in other taxonomic groups, such as insects, plants, and birds [75,76,77,78].

### 3.3. The Impact of Repeated Sequences on the Evolution of the W Chromosome in Caenophidian Snakes: Heterochromatinization Versus Heteromorphism

Since the early 1970s, it has been proposed that heterochromatinization, rather than the emergence of structural changes, represents the first stage in the differentiation of the W chromosomes in snakes [79]. Since then, several studies have been conducted to explore its genomic content [19]. The growing number of more advanced molecular investigations is leading to the reevaluation of previously accepted assumptions about sex chromosomes in snakes [6,21,80]. This exemplifies that we now recognize male heterogamy in primitive taxa, such as boas and pythons, which were previously thought to possess poorly differentiated ZZ/ZW systems comparable to those in birds [21,36].

Likewise, the sex chromosomes of advanced snakes have been characterized as derived, exhibiting a high degree of differentiation and possessing highly heterochromatic W chromosomes, traits considered synapomorphic of the clade [6,80,81]. Nonetheless, the architecture and development of sex chromosomes in snakes are far more complex than previously thought [21]. We observed that both *P. mucosa* and *C. ornata* had markedly distinct sex chromosomes, distinguished by a highly heterochromatic W chromosome enriched by repetitive sequences (Figure 1g,h,k,l and Figure 4). In contrast to the Z chromosome, the W chromosome of *P. korros* also exhibits a substantial amount of heterochromatin beyond the centromere, as well as a differential buildup of repeated sequences (Figure 1c,d). These sequences include the satDNAs PkoSat02-245 and PkoSat05-166, as well as the (AG)n, (AGAT)n, and (ATCC)n motifs (Figure 2 and Figure S3). Thus, heteromorphic sex chromosomes with accumulated repetitions may represent only a later stage in the evolution of sex chromosomes [82,83]. Despite these differences that we can analyze between Z and W chromosome of *P. korros*, intraspecific CGH was unable to identify genomic differences between the sex chromosomes, and this is due to the limitations of the technique, such as limited resolution or difficulty in hybridizing repetitive sequences, which are gaps that were filled by the other hybridizations and C-banding. On the other hand, the results obtained by interspecific CGH are corroborated by the genomic differences found in the sex chromosomes of *P. korros* and *P. mucosa*, with the satellite and microsatellite hybridization pattern highlighting an advanced differentiation between these two sister species.

An opposite scenario is found in *F. flavipunctatus*, which displays a fully euchromatic W chromosome, without any differential accumulation of microsatellites (Figure 1o,p and Figure 4). The scarcity of microsatellite accumulation on the sex chromosomes of this species is similar to the situation observed in the homomorphic sex chromosomes of more basal snakes [32,82,83], and could indicate an early stage of sex chromosome differentiation, despite the heteromorphism. However, this scenario is not restricted to a single species, and a similar situation has been observed in *Spilotes sulphureus* [33]. These results suggest that heteromorphism, the amount of heterochromatin, and the evolutionary degradation of sex snake chromosomes likely evolved independently, driven by yet unknown divergent processes [36,83,84,85]. This is a significant viewpoint for understanding the evolution of chromosomes since it suggests that these three factors should be treated independently. Previously, ref. [83] proposed that the accumulation of repetitive sequences is directly associated with the heterochromatization of the W chromosome; however, it is not always related to its heteromorphism. This phenomenon, although essential to chromosomal differentiation, is not restricted to particular events.

Snakes’ sex chromosomes appear to have inherited sequences from their common ancestor, subsequently influenced by diverse evolutionary pressures, leading to distinct patterns of nucleotide amplification or loss [6,80,81]. For example, the (AG)n motif was detected on the sex chromosomes of all examined snakes. The conservation of this pattern across various species indicates its potential inheritance from a common ancestor, serving a useful role in sexual differentiation, as previously reported in other reptilian taxa [36]. The absence of homology between the sex chromosomes of snakes and lizards suggests that the microsatellites common to both have experienced independent amplification events during their evolutionary history, as illustrated in Figure 6. Our study therefore supports the hypothesis of [36], which posits that sequences evolved independently in distinct reptilian lineages. An exemplary instance is the AAGG motif, which is common to certain snakes and *P. vitticeps*.

### 3.4. Genetic Content of Sex Chromosomes in Lizards and Their Relationship to the Genus Ptyas

BAC and sex chromosome probes derived from two Australian lizards [named *P. vitticeps* (Agamidae) and *V. acanthurus* (Varanidae)] were in situ mapped in the two *Ptyas* to investigate the possibility of homology between reptile species. Previous studies suggest that the W chromosome in snakes shares homology with the sex chromosomes of other amniotes [43]. Furthermore, the presence of homologous regions on several chromosomes in different species supports the idea that snake sex chromosomes evolved through chromosomal rearrangements mediated by repetitive sequences [43]. However, we did not find any positive signals. These results align with the current view that reptiles—a paraphyletic group of ectothermic amniotes—have variable and rapidly evolving sex determination and do not follow unique pathways of evolution as proposed for birds and mammals [86,87,88].

## 4. Materials and Methods

### 4.1. Sampling, Mitotic Chromosomal Preparations, and C-Banding

Table 1 summarizes the origin of the samples, the number of analyzed individuals, and their sex. *In vitro* leukocyte cultures were employed to obtain chromosomal preparations [89]. Constitutive heterochromatin was identified by the C-banding method [90]. All the procedures followed ethical protocols approved by the Institutional Animal Care and Use Committee of Khon Kaen University, based on the Ethics of Animal Experimentation of the National Research Council of Thailand (ACUC-KKU-90/60). Cell suspensions from *Pogona vitticeps* and *Varnaus acanthurus* were used from the University of Canberra chromosome collection previously obtained by [91,92]. No animals were seriously harmed, and all free-living individuals were released back to their respective collection sites. The authors complied with ARRIVE guidelines.

### 4.2. DNA Extraction and Genome Sequencing

The whole genomic DNAs (gDNAs) from males and females of *P. korros, P. mucosa, C. ornata,* and *F. flavipunctatus* were extracted by the standard phenol–chloroform method [93]. One male and one female of *Ptyas korros* were subjected to low-pass shotgun sequencing using the BGISEQ-500 platform at BGI Shenzhen (Shenzhen, China). The sequencing yielded 2.41 Gb and 2.45 Gb of data for the male and female, respectively (SRA accession numbers: SRR32267813 and SRR32267814).

### 4.3. Bioinformatic Analyses: The Characterization of Ptyas korros Satellitome

We independently characterized the satellite DNA catalog in *P. korros* of males and females. Initially, we quality-filtered the libraries using Trimmomatic [94]. Next, a subset of 2 × 500,000 reads was randomly sampled and analyzed for each library using TAREAN. All identified putative satellite DNAs were then retrieved and removed from the original dataset with DeconSeq [95]. Then, a new subset of 2 × 500,000 reads was extracted from the remaining sequences and subjected to another TAREAN iteration. This process was repeated iteratively until no additional satellite DNAs were identified. Subsequently, we screened and filtered out other repetitive DNA elements, such as multigene families. Finally, we grouped the sequences into variants (>95% similarity), families (80–95% similarity), and superfamilies (50–80% similarity) using pairwise alignments with MUSCLE [96].

### 4.4. Estimating the Abundance and Diversity of satDNAs

The genomic abundance of each satellite DNA was determined by calculating the proportion of mapped reads relative to the total number of analyzed nucleotides. This analysis used RepeatMasker [97] with the “cross-match” search engine. A dataset of 2 × 5,000,000 reads was randomly selected and aligned against the satellite DNA catalog of *P. korros* using a custom python script (https://github.com/fjruizruano/ngs-protocols/blob/master/repeat_masker_run_big.py, accessed on 10 December 2024). Additionally, genetic distances were estimated with the RepeatMasker, using the calcDivergenceFromAlign.py script. To visualize the divergence patterns among satellite DNA families, we generated repeat landscapes based on the Kimura-2-parameter model. All identified satDNAs were designated as PkoSatDNAs, sequentially numbered from the most to least abundant in the genome, as suggested by [98].

### 4.5. Primer Design, DNA Amplification, and Preparation of satDNA Probes

We designed primers for the six satDNAs we have isolated. The amplification procedure consisted of 34 cycles, starting with an initial denaturation at 95 °C for 45 s, followed by a ringing step at 58–64 °C for 30 s, an extension phase at 72 °C for 1 minute, and a final extension at 72 °C for 7 min. Each satDNA was amplified using 10 ng of female whole genomic DNA as a template. To identify *P. korros* satDNAs in other snake species, PCR was carried out using their genomic DNA as a template under the same conditions used for *P. korros*.

All satDNAs were labeled using a nick translation kit from Jena Bioscience (Jena, Germany) incorporating the fluorophore Atto488-dUTP or Atto550-dUTP according to the instructions in the manufacturer’s manual.

### 4.6. Microsatellite and Telomeric (TTAGGG)n Probes

We employed a set of microsatellites to investigate the role of repetitive sequences in the genomes of snake species. Five microsatellites, identified by [68] as strong candidates for association with sex chromosomes in reptiles, were analyzed: [(AGAT)n, (AAT)n, (AG)n, (ATCC)n, and (AAGG)n]. These sequences were labeled with Cy3 fluorophore during synthesis (VBC Biotech, Vienna, Austria) and also used in the hybridization experiments. Telomeric (TTAGGG)n sequences were detected using the Telomere PNA Fish Cy3 Kit (DAKO/Agilent, Santa Clara, CA, USA).

### 4.7. Comparative Genomic Hybridization (CGH): Experimental Design and Probe Preparation

Firstly, an intraspecific comparison in *P. korros* was performed to examine the extent of genetic differentiation between the sexes. For this, whole genomic DNAs (gDNAs) from males and females of *Ptyas korros* were labeled in green and red, respectively, with Atto550-dUTP and Atto488-dUTP, using nick-translation (Jena Biosciences, Jena, Germany). The labeled whole genomic DNAs for the sexes was coprecipitated with 20 μg of glycogen as a carrier, dissolved in 15 μL of hybridization buffer, and hybridized against the complement of the female chromosome following the FISH method, increasing the residence time at 37 °C for 3 days. No shared repetitive sequence blocking was used, following the protocol of [99]. For the second set of experiments, we performed interspecific comparisons between *P. korros* and *P. mucosa* to investigate the degree of differentiation of their W chromosomes. The methodology used was the same as for the intraspecific analysis, labeling the gDNA of *P. korros* females with Atto550-dUTP and the gDNA of *P. mucosa* females with Atto488-dUTP and hybridizing them against the female chromosomes of *P. korros*.

### 4.8. Investigation of Sex Chromosome Homology Between Snakes and Lizards

In our study, we also analyzed the putative homologies between the sex chromosomes of the bearded dragon (*P. vitticeps*) and the *Ptyas* species using two BAC clones from *P. vitticeps*, specifically pv03L07 and pv116G15, as described by [37]. These clones were selected due to their location on the Z and W chromosomes of the donor species. The probes were labeled with orange (pv3L7) and green (pv116G15) fluorophores using 0.5–1 µg of each BAC clone via nick translation (Abbott Molecular, Botany, NSW, Australia), following the manufacturer’s protocol with modifications. The labeled probes were incubated at 15 °C for 1 h and 45 min and then heated to 70 °C to terminate the reaction. They were subsequently coprecipitated with glycogen and 100% ethanol and stored at −20 °C overnight. The next day, the probes were centrifuged and resuspended in a hybridization buffer. Metaphase chromosomes from *P. vitticeps* were used as positive control.

Probes targeting the W and Z chromosomes of *V. acanthurus* [100] were also hybridized with chromosomes from the two *Ptyas* species and *P. vitticeps*.

### 4.9. Fluorescence In Situ Hybridization (FISH)

All FISH experiments were essentially carried out according to the protocol described in [101]; for the CGH experiments, we used the methodology described in [102].

### 4.10. Microscopic Analyses and Image Processing

At least 30 metaphase spreads per individual were analyzed to confirm the 2n, karyotype structure, FISH, and CGH results. Images were captured using an Olympus BX50 microscope (Olympus Corporation, Ishikawa, Japan), with CoolSNAP, and the images were processed using Image-Pro Plus 4.1 software (Media Cybernetics, Silver Spring, MD, USA). The karyotypes were arranged based on decreasing size (macro and microchromosome) since this configuration better reflects the predominant chromosomal organization observed in snakes and facilitates the comparison with previous studies.

## 5. Conclusions

The findings of our study offer significant contributions to the understanding of the evolution of the ZW system in snakes. The analysis revealed that, even in homomorphic sex chromosomes, such as those observed in *C. ornata* and *Ptyas* species, the W chromosomes can exhibit remarkable molecular variations, with unique repetitive sequences and being highly heterochromatic. In addition, it was observed that, despite the phylogenetic proximity between the species of the genus *Ptyas*, there was a low degree of sharing of satellite DNA sequences, suggesting independent and adaptive evolutionary paths for each species. Finally, snake ZW sex chromosomes have been shown to have differentiated significantly, acquiring unique characteristics in relation to their sister group, the lizards. These findings are essential for advancing the understanding of reptile evolution and provide new bases for future investigations into the molecular mechanisms that have shaped the genetic diversity of these animals. This study is part of a series of further cytogenetic and genomic studies focusing on the sex chromosome evolution in vertebrates.

## Figures and Tables

**Table 1:** Species, origin, sample size (N), and sex of the analyzed individuals.

Species (Abbreviation, When Used)	Origin of Samples	N
*Ptyas korros* (PKO)	Khon Kaen, Thailand	02♀; 01♂
*Ptyas mucosa* (PMU)	Khon Kaen, Thailand	02♀; 02♂
*Chrysopelea ornata* (COR)	Khon Kaen, Thailand	01♀; 01♂
*Fowlea flavipunctatus* (FFL)	Khon Kaen, Thailand	01♀; 01♂
*Notechis scutatus* (NSC)	Canberra, Australia	01♀
*Pogona vitticeps* (PVI)	Canberra, Australia	01♀
*Varanus acanthurus* (VAC)	Canberra, Australia	02♀

## Data Availability

The datasets generated during and/or analyzed during the current study are available from the corresponding author upon reasonable request. The datasets generated and analyzed during the current study are available in the GenBank repository under the accession numbers PV358982–PV358987.

## Supplementary Materials

The following supporting information can be downloaded at: https://www.mdpi.com/article/10.3390/ijms26104540/s1.

## Abbreviations

The following abbreviations are used in this manuscript:

satDNA	Satellite DNA
BAC	Bacterial artificial chromosome
rDNA	Ribosomal DNA
ITS	Interstitial sequences
PKO	*Ptyas korros*
PMU	*Ptyas mucosa*
COR	*Crysopelea ornata*
FFL	*Fowlea flavipunctatus*
RUL	Repeat unit length
PKOsatDNA	Satellite DNA from *Ptyas korros*
PCR	Polymerase chain reaction
FISH	Fluorescent in situ hybridization
gDNA	whole genomic DNA
DAPI	4′,6-diamidino-2-phenylindole
CGH	Comparative genomic hybridization
