# Supplementary material for: Evolution of ZW Sex Chromosomes in Ptyas Snakes (Reptilia, Colubridae): New Insights from a Molecular Cytogenetic Perspective
Source: Int J Mol Sci. 2025 May 9;26(10):4540. doi: 10.3390/ijms26104540 (PMC12111239; doi:10.3390/ijms26104540)
Supplement: Supplementary file 1 [file ijms-26-04540-s001.zip › ijms-3580271-SI.pdf]

Supplementary Materials

Table S1: Main characteristics of the *Ptyas korros* satDNAs.

satDNA family	Rul	Abundance (F)	Abundance (M)	Abundance (F/M)	A+T (%)
PkoSat1-168	168	0,001149156	0,000841797	1,365122953	63,0952381
PkoSat2-245	245	0,000731083	0,000759606	0,962450493	56,73469388
PkoSat3-324	324	0,000572737	0,000611321	0,936885308	63,27160494
PkoSat4-149	149	0,000216136	0,000114426	1,888880659	63,08724832
PkoSat5-166	166	0,000152454	0,000166577	0,915216338	62,04819277
PkoSat6-150	150	0,000131901	0,000164344	0,802592203	64,66666667

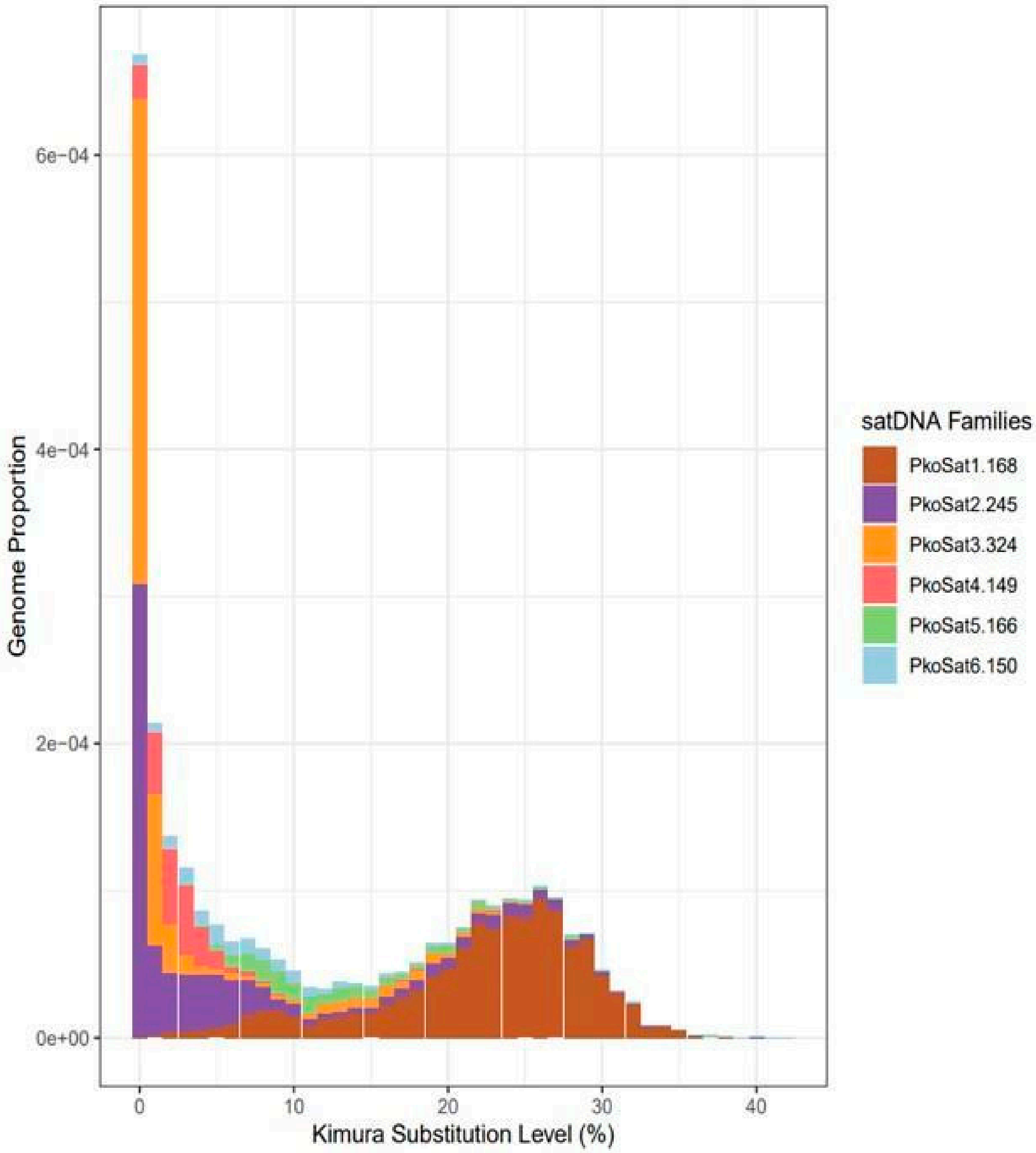

Figure S1. Repeat landscapes illustrating the distribution and divergence of all PkoSatDNA families identified in *Ptyas korros* genome.

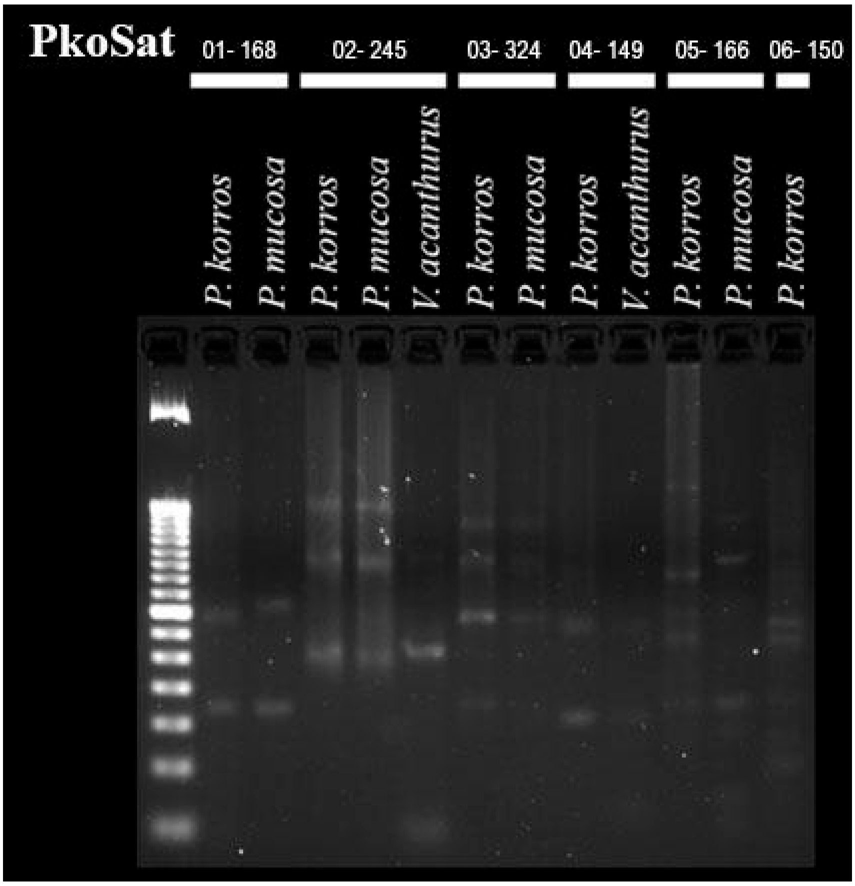

Figure S2: Electrophoresis gel showing the amplification pattern of all the satellites in *Ptyas korros* and those amplified in the congeneric species.

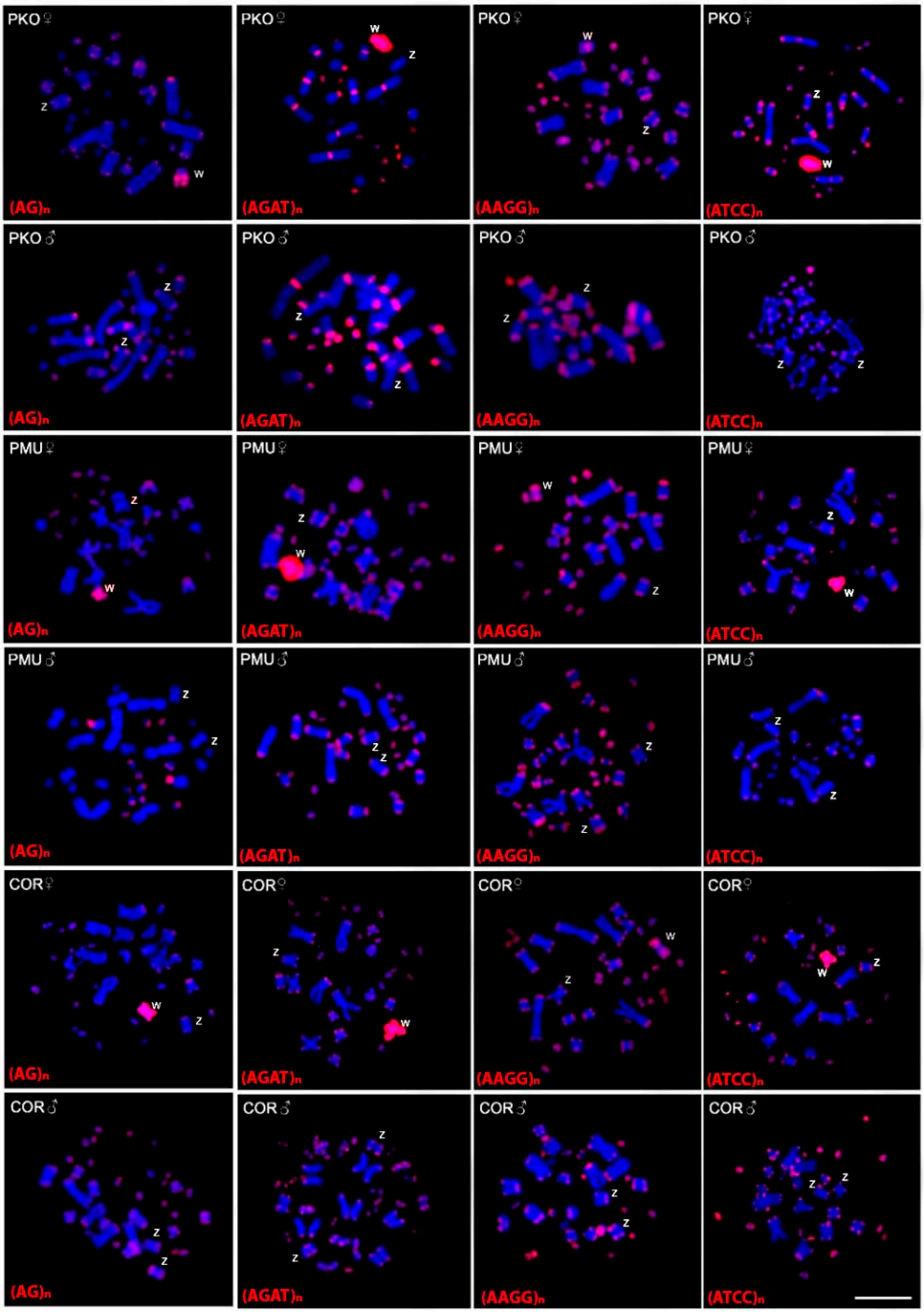

Figure S3: Microsatellite sequences (identified in red in the lower left corner) hybridized in males and females of *P. korros* (PKO), *P. mucosa* (PMU) and *Crysopelea ornata* (COR). Scale bar = 10 µm.

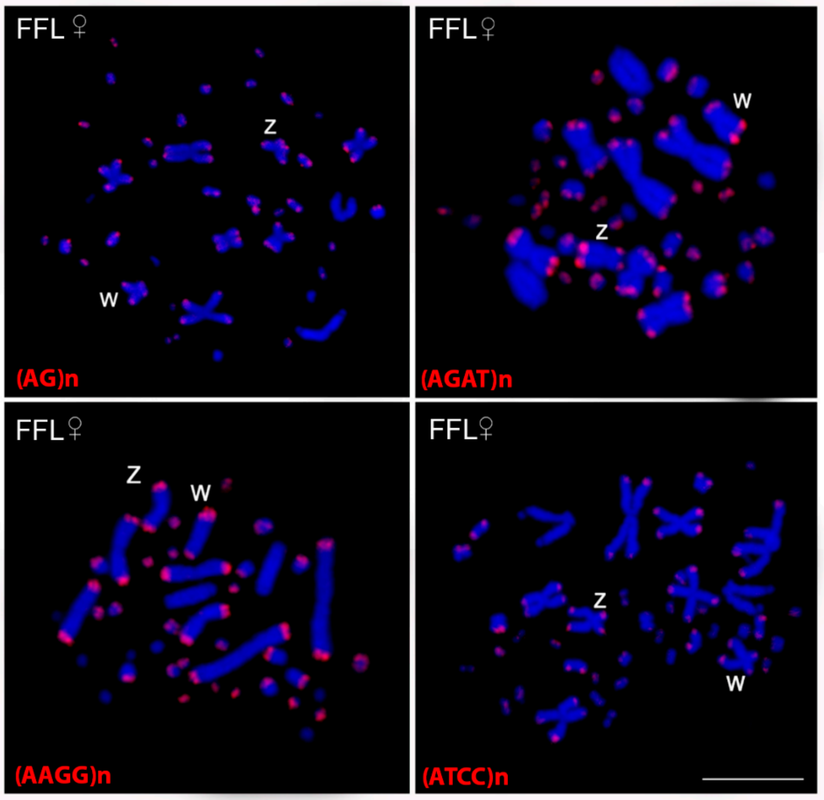

Figure S4: Female metaphase plates of *Fowlea flavipunctatus* (FFL) hybridized with distinctive microsatellite sequences (identified in red in the lower right corner). Scale bar = 10 µm.

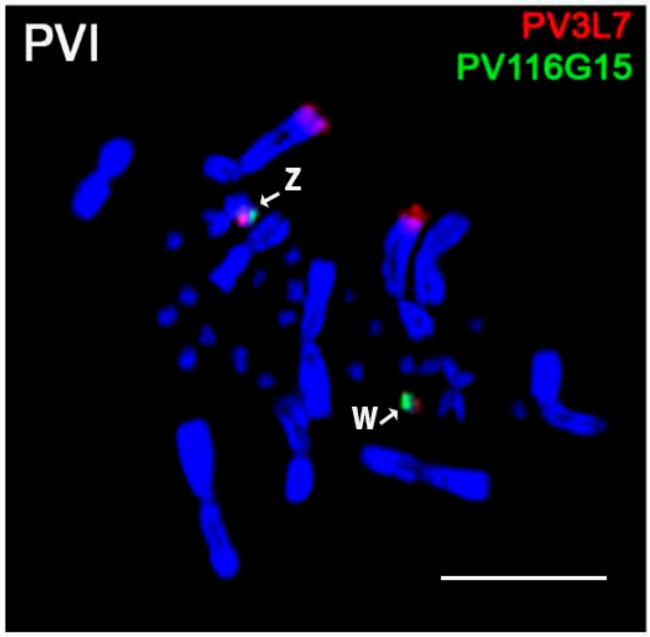

Figure S5: Female metaphase plates of *Pogona vitticeps* (PVI) hybridized with the BAC probes PV3L7 (red) and PV116G15 (green). Scale bar = 10 µm.

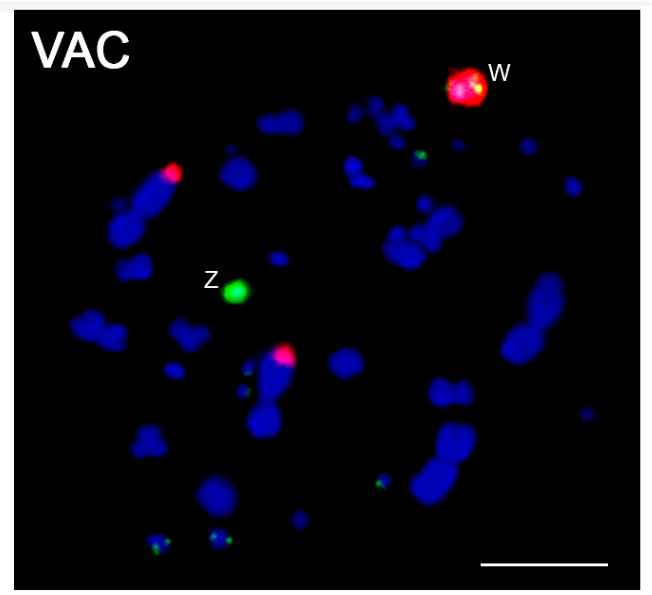

Figure S6: Sex-linked probes of Z (green) and W (red) chromosomes in the donor species *Varanus acanthurus* (VAC). Scale bar = 10 µm.
